# UNDERSTANDING CULTURAL NARRATIVES OF DEMENTIA: TASKS AND TOOLS FOR HUMANITIES SCHOLARSHIP

**DOI:** 10.1093/geroni/igac059.962

**Published:** 2022-12-20

**Authors:** Nancy Berlinger

**Affiliations:** The Hastings Center, Garrison, New York, United States

## Abstract

Humanities scholarship on dementia has long focused on the depiction of dementia in literature, film, and other genres. Recent research on neurodiversity includes humanistic scholarship on creativity within dementia. It is time for interdisciplinary humanities scholarship to focus on narratives of dementia that circulate within aging societies, are embedded in policy, and shape experiences of typical people living with dementia or providing dementia care. This paper argues for the normative importance of studying values-laden cultural narratives, recognizing competing or evolving narratives within a society, and demonstrating how to reframe flawed narratives beyond necessary attention to ageist and ableist language. It presents examples of approaches to social narrative analysis; describes tools and training that could be integrated into humanities scholarship on dementia and aging, and considers the potential role of social narrative analysis in articulating and launching policy ideas for aging societies.

